# Whole tumor kinetics analysis of ^18^F-fluoromisonidazole dynamic PET scans of non-small cell lung cancer patients, and correlations with perfusion CT blood flow

**DOI:** 10.1186/s13550-018-0430-4

**Published:** 2018-08-01

**Authors:** Daniel R. McGowan, Michael Skwarski, Bartlomiej W. Papiez, Ruth E. Macpherson, Fergus V. Gleeson, Julia A. Schnabel, Geoff S. Higgins, John D. Fenwick

**Affiliations:** 1 Department of Oncology, University of Oxford, Oxford, OX3 7DQ UK; 2 Radiation Physics and Protection, Oxford University Hospitals NHS Foundation Trust, Oxford, UK; 3 Institute of Biomedical Engineering, Department of Engineering Science, University of Oxford, Oxford, UK; 4 Big Data Institute, Li Ka Shing Centre for Health Information and Discovery, University of Oxford, Oxford, UK; 5 Department of Radiology, Oxford University Hospitals NHS Foundation Trust, Oxford, UK; 6 School of Biomedical Engineering and Imaging Sciences, King’s College London, London, UK; 7 Department of Oncology, Oxford University Hospitals NHS Foundation Trust, Oxford, UK; 8 Institute of Translational Medicine, University of Liverpool, Liverpool, UK

**Keywords:** FMISO, NSCLC, Dynamic PET, Kinetics analysis, Perfusion CT

## Abstract

**Background:**

To determine the relative abilities of compartment models to describe time-courses of 18F-fluoromisonidazole (FMISO) tumor uptake in patients with advanced stage non-small cell lung cancer (NSCLC) imaged using dynamic positron emission tomography (dPET), and study correlations between values of the blood flow-related parameter *K*_1_ obtained from fits of the models and an independent blood flow measure obtained from perfusion CT (pCT).

NSCLC patients had a 45-min dynamic FMISO PET/CT scan followed by two static PET/CT acquisitions at 2 and 4-h post-injection. Perfusion CT scanning was then performed consisting of a 45-s cine CT.

Reversible and irreversible two-, three- and four-tissue compartment models were fitted to 30 time-activity-curves (TACs) obtained for 15 whole tumor structures in 9 patients, each imaged twice. Descriptions of the TACs provided by the models were compared using the Akaike and Bayesian information criteria (AIC and BIC) and leave-one-out cross-validation. The precision with which fitted model parameters estimated ground-truth uptake kinetics was determined using statistical simulation techniques. Blood flow from pCT was correlated with *K*_1_ from PET kinetic models in addition to FMISO uptake levels.

**Results:**

An irreversible three-tissue compartment model provided the best description of whole tumor FMISO uptake time-courses according to AIC, BIC, and cross-validation scores totaled across the TACs. The simulation study indicated that this model also provided more precise estimates of FMISO uptake kinetics than other two- and three-tissue models.

The *K*_1_ values obtained from fits of the irreversible three-tissue model correlated strongly with independent blood flow measurements obtained from pCT (Pearson *r* coefficient = 0.81). The correlation from the irreversible three-tissue model (*r* = 0.81) was stronger than that from than *K*_1_ values obtained from fits of a two-tissue compartment model (*r* = 0.68), or FMISO uptake levels in static images taken at time-points from tracer injection through to 4 h later (maximum at 2 min, *r* = 0.70).

**Conclusions:**

Time-courses of whole tumor FMISO uptake by advanced stage NSCLC are described best by an irreversible three-tissue compartment model. The *K*_1_ values obtained from fits of the irreversible three-tissue model correlated strongly with independent blood flow measurements obtained from perfusion CT (*r* = 0.81).

**Electronic supplementary material:**

The online version of this article (10.1186/s13550-018-0430-4) contains supplementary material, which is available to authorized users.

## Background

The radiotracer ^18^F-fluoromisonidazole (FMISO) diffuses passively into cells, where it is reduced and irreversibly bound in hypoxic environments. Thus, positron emission tomography (PET) imaging of FMISO uptake can be used to localize hypoxic tumor subvolumes [1–3]. The degree of hypoxia can be estimated either from uptake levels seen in single FMISO images collected 2–4 h after tracer injection [4], or from analysis of the kinetics of FMISO uptake in dynamic sequences of PET images (dPET). The kinetic analysis provides fitted values of model rate-constants related to blood flow and FMISO transport and intracellular binding and can be performed at the whole tumor level or voxel-by-voxel. For head-and-neck cancers, it has generated indices that correlate with radiotherapy (RT) outcomes [5].

Blood flow is often imaged using a perfusion CT (pCT) technique first proposed in 1980, in which iodine containing contrast is injected as a bolus through a venous cannula, and its passage through the patient is dynamically imaged and kinetically analyzed on the assumption that iodine concentrations within tissues are linearly proportional to changes in measured CT attenuation [6]. Blood flow measures obtained from pCT have been found to correlate with perfusion measurements obtained from dPET imaging of ^15^O-water uptake [7], which in turn were correlated with kinetics indices obtained from compartment modeling of the first 2 min of ^18^F-fluorodeoxyglucose (FDG) dPET scans [8].

In this study, we investigate which of several compartment models best describes PET-imaged time-courses of FMISO uptake in whole NSCLC tumors, and we identify the model whose fits to the time-course data provide the most precise estimates of tracer kinetics rate-constants according to statistical simulations. Correlations are determined between perfusion measures obtained directly from the PET FMISO kinetics model fits and independently from pCT.

## Methods

### PET and pCT image acquisition and processing

In a pre-clinical study, the investigational drug buparlisib (Novartis) reduced tumor hypoxia in vivo [9]. A clinical trial (BKM120) completed in Oxford (NCT02128724) has the primary aim of determining the maximum tolerated dose of buparlisib in non-small cell lung cancer (NSCLC) patients treated palliatively using radiotherapy, and the secondary goal of validating the pre-clinical results in these patients, who are imaged using FMISO PET at baseline and 7 days after administration of buparlisib without any other intervention. The study has been approved by the local ethics committee and signed informed consent obtained from all patients.

Patients were imaged supine with their arms by their side using a GE Discovery 690 PET/CT scanner (GE Healthcare). They were injected with 370 MBq FMISO 30 s into PET imaging, which continued for 45 min and resumed for 10 min intervals at 2 and 4-h post-injection. Prior to each PET acquisition, a CT scan was performed for localization and PET attenuation correction. PET images were reconstructed using a time-of-flight ordered subset expectation maximization algorithm (VPFX, GE Healthcare). The first 45 min of data were binned into two parallel time sequences, S1 (1 s × 30 s, 12 s × 5 s, 6 s × 10 s, 5 s × 30 s, 10 s × 60 s, 6 s × 300 s) and S2 (1 s × 30 s, 60 s × 1 s, 12 s × 10 s, 3 s × 30 s, 10 s × 60 s, 6 s × 300 s), and reconstructed as images on a matrix of 5.5 mm^3^ × 5.5 mm^3^ × 3.3 mm^3^ voxels. Data collected during the two later 10-min intervals were processed as single frames [10].

pCT scanning was performed immediately after PET/CT imaging concluded at 4-h post-injection of FMISO, with patients set up in the same position on the same PET/CT scanner. Initially a pre-contrast CT scan (helical mode, 120 kV, smart mA, 32 noise index) was carried out to determine the region over which the pCT data would be collected. Then pCT scanning commenced (120 kV, 60 mA), collecting one 3D image in each of 45 consecutive seconds, over an axial length of 40 mm corresponding to the CT detector width. During pCT scanning 70 mL contrast (Omnipaque 300) was injected at 5 mL/s, followed by 25 mL water at 5 mL/s, with patients instructed to hold their breath at inspiration for as long as possible, breathing out very slowly if necessary.

For each patient, the primary tumor, involved nodes, and metastases were outlined on the PET/CT images by an experienced radiologist, and a blood region was defined within the central part of the descending aorta on five or more consecutive PET axial slices [11]. These were outlined on the CT images with the patient’s prior contrast-enhanced CT imaging used to assist in determining tumor regions. Time-activity curves (TACs) representing time-courses of mean FMISO tracer activity concentrations within each tumor volume-of-interest (VOI) and the blood region were obtained from PET sequences S1 and S2 respectively. Activity data from the 10-min frames collected at 2 and 4 h post-injection were appended to the TACs. A total of 30 whole-volume tumor FMISO uptake TACs, obtained for 15 volumes-of-interest (9 primary tumors, 5 involved nodes, and 1 metastasis) in 9 patients, each imaged twice were studied (Table 1). Using a standard tumor to blood ratio of 1.4 (on the static images four hours post-injection), all of these whole tumor volumes had a number of voxels within them which could be considered to be hypoxic [4]. No direct oxygen measurements of the tumors were made in this trial.

**Table 1:** Details of the 30 whole tumor TACs analyzed

Patient	VOIs and TAC reference numbers in images taken pre/post drug administration
1	Primary (1/2)
2	Primary (3/4), metastasis (5/6)
3	Primary (7/8)
4	Primary (9/10)
5	Primary (11/12)
6	Primary (13/14), node (15/16)
7	Primary (17/18), node (19/20)
8	Primary (21/22), node 1 (23/24), node 2 (25/26), node 3 (27/28)
9	Primary (29/30)

**Fig. 1:**
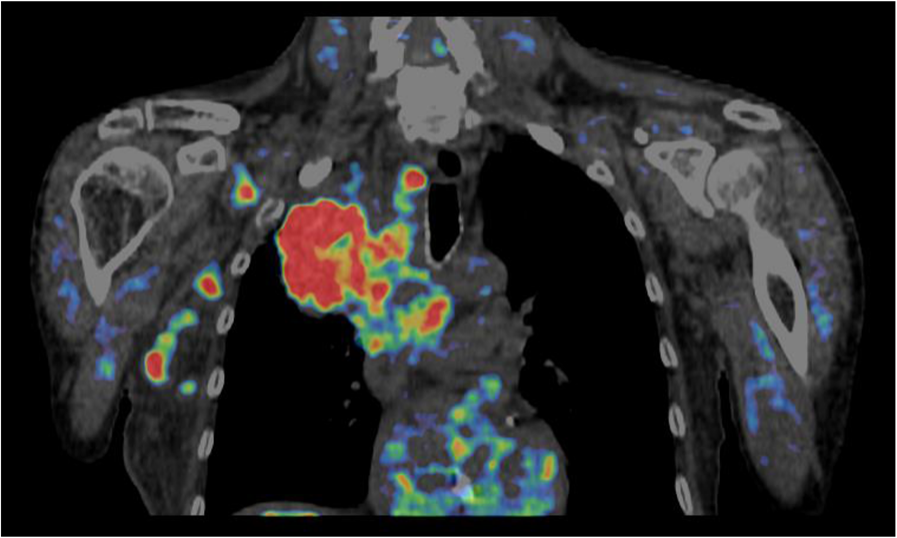
An example coronal FMISO PET image fused with the corresponding CT displayed on a tumor to blood ratio (TBR) color scale. Red regions depict a TBR > 1.4 indicating hypoxia, and no visible PET depicts a TBR < 1, indicating normoxia [4]

Using Hermes Hybrid Viewer software (Hermes Medical Solutions AB, Sweden), the CT images obtained at PET/CT were rigidly registered with the CT images collected just before pCT, allowing outlines of the primary tumors defined on the PET/CT to be transferred to the pCT scans. An example of a FMISO PET/CT image 4 h post-injection is shown in Fig. 1.

### PET kinetics analysis and model fitting

**Fig. 2:**
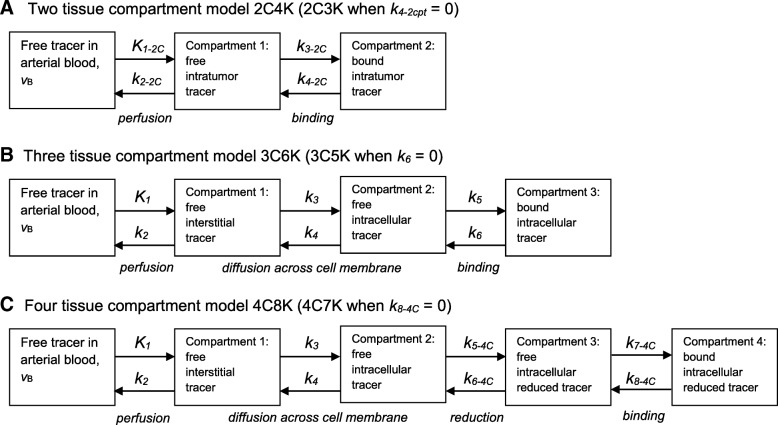
**a** Two-, **b** three-, and **c** four-tissue compartment models used to analyze the dynamic PET data. Flow rates from one compartment to another are defined by rate-constants (*k* values), tracer concentrations and compartment volumes [12]*.* The fraction of the tumor volume occupied by blood is denoted in each model as *ν*_*B*_

Several methods have been used to analyze dPET data, the most common being compartment modeling [12]. Figure 2 illustrates reversible two-, three-, and four-tissue linear compartment models which we have fitted to time-courses of tumor tracer uptake [13–15]. FMISO binding is generally considered irreversible, and therefore, alongside reversible models, we have also studied irreversible models in which the rate-constant describing movement of bound to unbound tracer is set to zero. The compartment model developed by Casciari et al. [2] attempts to reflect the chemical processes that occur for FMISO uptake; however, this model has many fitting parameters, and so in this work, simpler compartment models have been investigated. We denote by *x*C*y*K a model comprising a linear chain of *x-*tissue compartments (excluding blood-borne tracer) and *y* rate-constants, and in order to associate rate-constants with particular models, we add the subscript *x*C to the names of rate-constants, except for those of the three-tissue compartment model.

Kinetics analysis was carried out using the PMOD system (PMOD Technologies) as described by McGowan et al. [10] In brief, an image-derived FMISO input function [16, 17] was obtained from the blood region outlined within the descending aorta, and a compartment model was fitted to the tumor TAC and input function data, generating fitted values of model rate-constants. Fitting was carried out by minimizing the weighted sum of squares between modeled and measured tumor uptake, using the Levenberg–Marquardt algorithm with weighting function

1 $$ {w}_i=\Delta {t}_i\ \exp \left(-\lambda {t}_i\right)/{C}_{PET}\left({t}_i\right) $$

where *t*_*i*_ and ∆*t*_*i*_ are the mid-time post-injection and duration of the *i*th of *T* frames, *λ* is the decay constant for ^18^F, and *C*_*PET*_ the measured PET activity concentration at time *t*_i_ [15, 17].

Model fitting was initiated from 100 randomly generated sets of starting values (suitably constrained), to attempt to reach global rather than local best fits [18]. Flux-constants were calculated from irreversible two-tissue compartment model (2C3K) fits as

2 $$ {k}_{flux-2C}=\kern0.5em \frac{K_{1-2C}\ {k}_{3-2C}}{k_{2-2C}+\kern0.5em {k}_{3-2C}} $$

and from irreversible three-tissue (3C5K) compartment model fits as

3 $$ {k}_{flux}=\kern0.5em \frac{K_1{k}_3{k}_5}{k_2{k}_4+{k}_2{k}_5+{k}_3{k}_5} $$

### Assessment of PET kinetic model fits

The Wald–Wolfowitz runs-test was used to determine the adequacy of descriptions of FMISO uptake TACs provided by compartment model fits [19, 20]. To further assess the relative abilities of the different models to describe the data, we used the Akaike information criterion (AIC) [21] corrected for small sample size [22], the Bayesian information criterion (BIC) [22], and leave-one-out cross-validation [23]. The cross-validation approach proceeded by fitting each model to the complete dataset minus one point, calculating the differences between the value of the omitted data-point and values predicted by the models, repeating this process sequentially *T* times leaving out a different data-point each time, and finally calculating the mean of the squared error of prediction (MSEP) for each model, the model with the lowest MSEP being considered best. These model selection methods use slightly different criteria to determine the model that best describes the data (all penalizing highly parameterised models which are likely to over fit the data), for completeness all have been included in this work.

A statistical simulation procedure was used to assess which model produced the most accurate and precise rate-constant estimates [10]. For this analysis, we used 3C5K and 3C6K model fits to the 30 measured whole tumor TACs to create noise-free “ground-truth” TACs binned into the same frame-lengths as the original data, and the parameter values of these fits were taken as ground-truth rate-constants. For each of the resulting 60 ground-truth TACs, 1000 noisy TACs were simulated by adding normally distributed random variables to the activity concentrations of the individual time-frames, the variances of the noise differing between frames according to the inverse of Eq. (1) and scaled to match noise-levels seen on the measured whole tumor TACs [10, 15, 24–26]. The average whole tumor scaling factor with the weighting factor used here was 0.6 ± 0.3 (one standard deviation). The simulated TACs were then fitted using the 2C3K, 2C4K, 3C5K, and 3C6K models.

The simulated noise introduces random uncertainties and systematic error (bias) into fitted parameter values, adding to any underlying bias that results from mismatches between the fitted models and the ground-truth models used to generate the simulated TACs. As described by McGowan et al. [10], for each of the 30 ground-truth TACs associated with each ground-truth model, individual biases in parameter values were determined from fits to the 1000 noise realizations, and then these bias estimates were combined to calculate the overall mean bias (*MB*) and variance of bias values (${\sigma}_B^2$) across the ground-truth TACs. The mean variance (${\sigma}_P^2$) was calculated for each parameter as the average of the parameter variances obtained for each of the 30 ground-truth TACs. Then, the *σ*_*B*_ and *σ*_*P*_ terms were combined to generate a total uncertainty, *σ*_*T*_, given by

4 $$ {\sigma}_T={\left({\sigma}_B^2+{\sigma}_P^2\right)}^{1/2} $$

For some fitted models, certain individual rate-constants are not uniquely related to any single ground-truth model parameter: for example, the processes described by the *K*_*1–2C*_ parameter of two-compartment models are split between rate-constants *K*_1_ and *k*_3_ in three-compartment models. For such rate-constants, therefore, only the *σ*_*P*_ values were calculated.

### Perfusion CT analysis and comparison with PET kinetic modeling

The small size of pCT image voxels (0.7 mm ×0.7 mm × 50 mm) makes parametric images of perfusion susceptible to movement [27], which can easily occur as it is difficult for patients to hold their breath for the full duration of pCT scanning. We therefore used a non-rigid image registration algorithm to pre-process the pCT data. The algorithm was based on the diffeomorphic demons approach, modified by use of normalized gradient fields (NGF) to handle intensity changes caused by contrast uptake. The registration algorithm uses a multi-resolution framework with three levels (128 × 128 × 8, 256 × 256 × 8, 512 × 512 × 8), the final spacing being equal to the original voxel spacing. The maximum number of iterations for each level was 25, and the standard deviation of the Gaussian smoothing kernel was 2.8 mm, 1.4 mm, and 0.7 mm at the different resolution levels. Further details have been provided by Papiez et al [27].

**Fig. 3:**
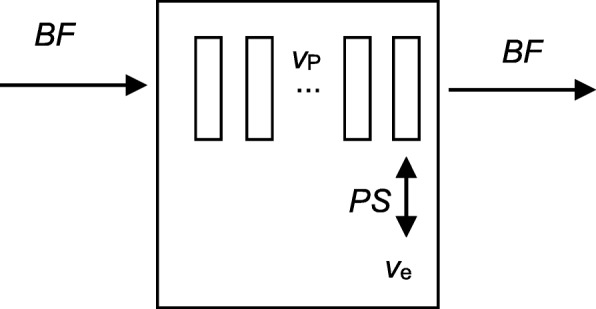
Schematic of the AATH model used to analyze the perfusion CT data: *BF* is the blood flow through the tumor, *v*_P_ the intravascular plasma volume fraction, *v*_e_ the extracellular extravascular space, and *PS* the permeability surface area product which defines the exchange rate between plasma and the extravascular extracellular space

The motion-corrected pCT data was then processed voxel-by-voxel using the commercial GE Perfusion 4D software (GE Healthcare, Milwaukee, USA). Voxel-by-voxel tumor blood flow information was obtained by fitting the Adiabatic Approximation to the Tissue Homogeneity (AATH) model [28] to the pCT TACs of each voxel, which describe the variation of voxel X-ray attenuation coefficient with time. Similarly to the compartment models used in the PET kinetics analysis, the AATH model describes the time-course of attenuation, and thus of iodine uptake, as the convolution of an iodine input function, obtained from a VOI drawn in the center of the descending aorta, with a residue function containing fittable parameters. A schematic of the AATH model is shown in Fig. 3: unlike the PET models, blood flowing through the tumor is considered to have a finite transit time, with contrast exchanged between intra and extravascular spaces only at the venous outlet.

**Fig. 4:**
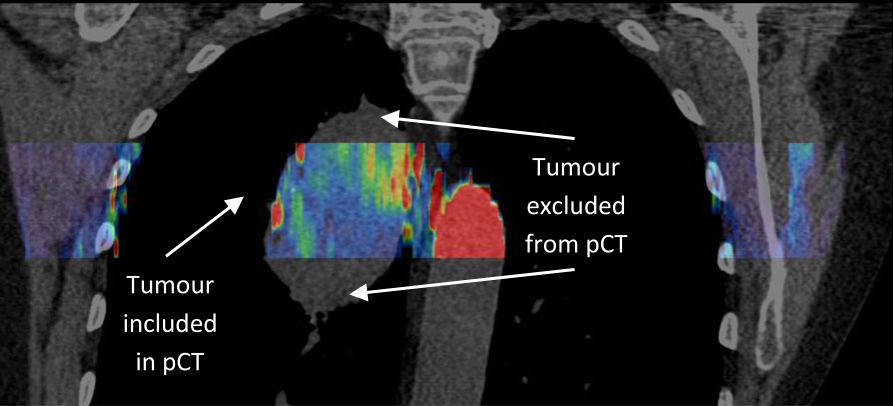
A coronal slice taken from one patient; the section in color indicates the volume over which the pCT was performed, while the underlying greyscale CT is from the pre-contrast CT

Voxel-by-voxel values of blood flow, *BF*, were taken from the resulting parametric images and averaged over tumor volumes. Then, the averaged *BF* values for each tumor volume were compared to measures obtained from PET kinetics analysis. When the whole tumor volume exceeded the 4-cm axial length of the pCT scans, as shown in Fig. 4, the PET kinetic analysis was repeated just for the tumor subvolume lying within the pCT field of view, allowing results obtained from pCT perfusion and PET kinetics analyses to be meaningfully compared.

The *K*_*1*_ rate-constant obtained from PET kinetic modeling is conceptually linked to *BF* via

5 $$ {K}_1= BF\times E $$

in which the extraction fraction *E* for a cylindrical capillary is given by [29, 30]

6 $$ E=1-\exp \left(- PS/ BF\right) $$

where *P* is capillary permeability, *S* the surface area per unit volume, and *PS* the permeability surface area product. For highly permeable tracers such as FMISO [31], *PS* is much greater than *BF* and the extraction fraction is close to 1. Consequently, *BF* measurements obtained from pCT scans should be approximately equal to *K*_1_ derived from FMISO PET kinetic modeling. We have therefore determined the Pearson *r* coefficients of correlation between pCT tumor mean *BF* values and *K*_1_ values obtained from 2C3K and 3C5K compartment model fits to the dPET data. Correlations were also evaluated between *BF* values and static FMISO uptakes at each time-point in the dynamic series of images, and between *BF* and the average FMISO uptakes over the first 2 min post-injection.

## Results

### Quality of compartment model fits to FMISO TACs

The numbers of whole tumor FMISO TACs for which fits of each compartment model passed the runs-test are listed in Table 2, together with total AIC, BIC, and MSEP scores for the different models summed over all TACs, and the numbers of TACs for which each model achieved the lowest scores. Runs-test results are presented individually for each whole tumor TAC in Additional file 1: Table S1 with corresponding AIC, BIC, and MSEP scores detailed in Additional file 2: Table S2.

**Table 2:** Summary of runs-test results and totaled AIC, BIC, and MSEP scores for compartment model fits to all 30 whole tumor FMISO TACs. Lowest totaled AIC, BIC, and MSEP scores are underlined, indicating the best model according to those measures

Model	2C3K	2C4K	3C5K	3C6K	4C7K	4C8K
Runs-test passes from fits to all 30 TACs						
*Runs*	0	6	25	26	30	30
Information criteria and cross-validation scores summed for all TACs						
*AIC*	13,970	8600	1544	1554	1557	1644
*BIC*	14,174	8836	1813	1840	1850	1945
*MSEP*	219	131	24.6	26.9	24.8	27.9
Numbers of TACs for which each model has the lowest scores						
*AIC*	0	0	21	3	6	0
*BIC*	0	0	22	3	5	0
*MSEP*	0	0	24	3	3	0

**Fig. 5:**
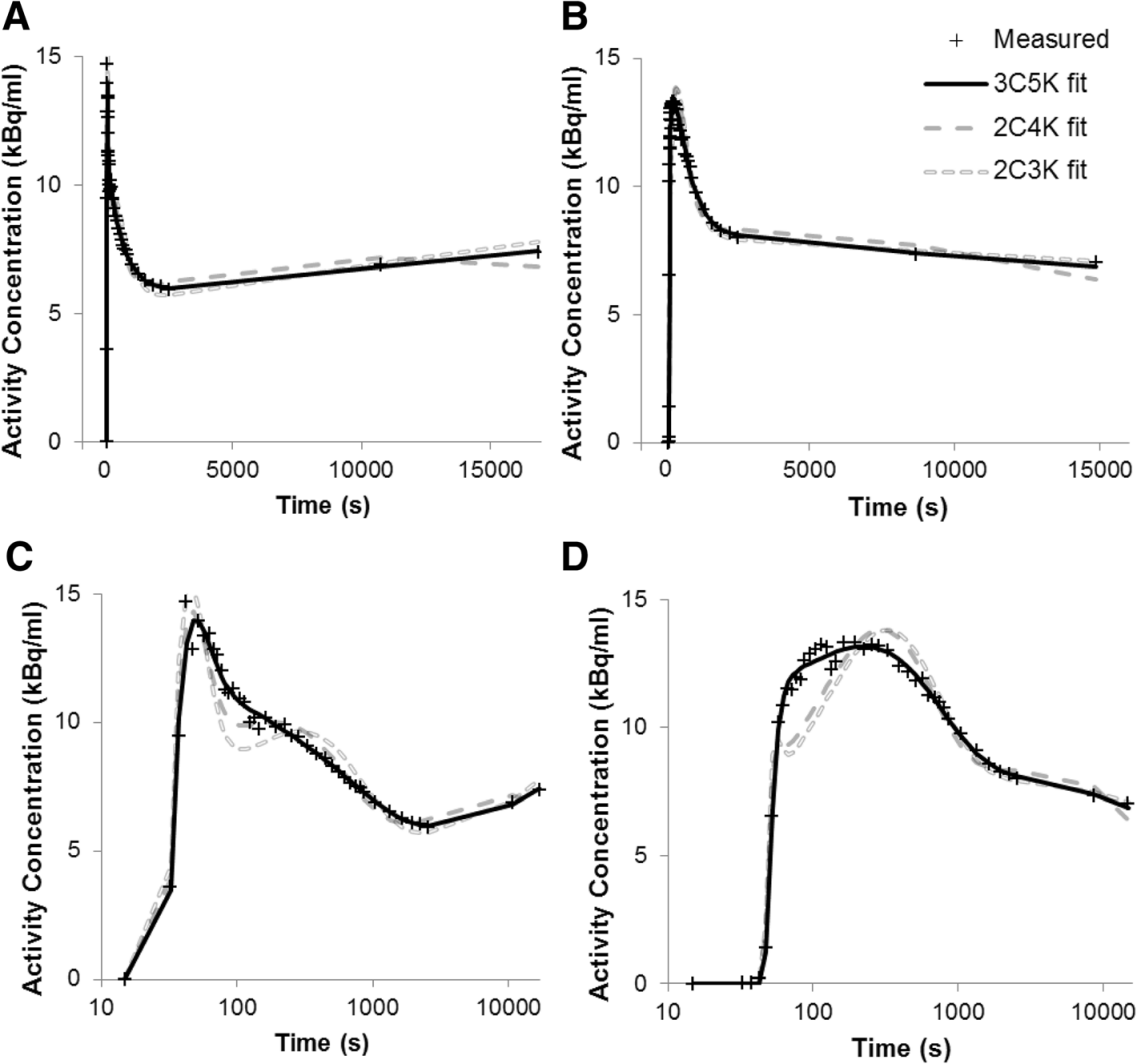
Fits of the 2C3K, 2C4K, and 3C5K models to FMISO TACs 4 (plots **a** and **c**) and 24 (**b** and **d**). Time post-injection is plotted on linear (**a**/**b**) and logarithmic (**c**/**d**) scales to enable the early part of the TAC and modeled TAC to be visualized

The three- and four-tissue models passed the runs-test for 83–100% of whole tumor TACs, whereas two-tissue model fits passed for only 0–20% [32]. Summed AIC, BIC, and MSEP scores were much lower for three- and four-tissue models than for two-tissue models, which lacked the flexibility to describe the data well. Fits of the 2C3K, 2C4K, and 3C5K models to two example TACs (4 and 24) are plotted in Fig. 5.

The irreversible three-tissue model, 3C5K, achieved the lowest AIC, BIC, and MSEP scores totaled over all tumor TACs, and the lowest scores for most (21–24) individual TACs. Individual scores were generally a little higher for the 3C6K, 4C7K, and 4C8K models, their additional complexity usually being unnecessary to describe the TAC data.

Table 3 lists estimates of parameter accuracy and precision obtained from the statistical simulations, which used fits of the 3C5K and 3C6K models to measured FMISO TACs to represent the ground-truth, the 3C5K model offering the best description of the TACs according to the AIC, BIC, and MSEP measures. Parameter values obtained from fits of the 2C4K model to simulated noisy TACs had large mean biases and total uncertainties, irrespective of which three-tissue ground-truth model was used. Mean biases and uncertainties of fitted 2C3K model parameters were not so large as for 2C4K, but were still considerably larger than those estimated for three-compartment models.

**Table 3:** Estimates of the accuracy and precision of parameter values obtained from fits of the 2C3K, 2C4K, 3C5K, and 3C6K models to whole tumor TACs simulated by adding whole tumor-level noise to the ground-truth, represented as 3C5K and 3C6K model fits to real TACs. Values of *MB*, *σ*_*B*_, *σ*_*P*_*,* and *σ*_*T*_ are shown for fitted parameters as percentages of the mean values of directly related ground-truth parameters in the 3C5K or 3C6K models. When no directly related parameter exists, *σ*_*P*_ is listed alone as a percentage of the mean fitted parameter value

Model fitted		Fitted model parameters						
Ground-truth 3C5K model								
2C3K	*v*_B–2C_	*K*_1–2C_	*k*_2–2C_			*k*_3–2C_		*k*_flux–2C_
*MB* (%)	47	**–**	**–**			−40		25
*σ*_*B*_ (%)	24	**–**	**–**			23		11
*σ*_*P*_ (%)	14	30	36			12		13
*σ*_*T*_ (%)	27	**–**	**–**			26		17
2C4K	*v*_B–2C_	*K*_1–2C_	*k*_2–2C_			*k*_3–2C_	*k*_4–2C_	*k*_flux–2C_
*MB* (%)	25	**–**	**–**			565	**–**	1014
*σ*_*B*_ (%)	20	**–**	**–**			700	**–**	1049
*σ*_*P*_ (%)	9	7	13			73	45	47
*σ*_*T*_ (%)	22	**–**	**–**			704	**–**	1050
3C5K	*v*_B_	*K*_1_	*k*_2_	*k*_3_	*k*_4_	*k*_5_		*k*_flux_
*MB* (%)	0	− 1	0	1	0	0		0
*σ*_*B*_ (%)	4	1	2	4	3	1		0
*σ*_*P*_ (%)	10	5	10	20	11	10		6
*σ*_*T*_ (%)	10	5	10	20	12	10		6
3C6K	*v*_B_	*K*_1_	*k*_2_	*k*_3_	*k*_4_	*k*_5_	*k*_6_	*k*_flux_
*MB* (%)	0	0	1	5	3	13	**–**	13
*σ*_*B*_ (%)	4	1	2	7	5	17	**–**	16
*σ*_*P*_ (%)	10	5	9	22	13	34	384	31
*σ*_*T*_ (%)	11	5	9	23	14	38	**–**	35
Ground-truth 3C6K model								
2C3K	*v*_B–2C_	*K*_1–2C_	*k*_2–2C_			*k*_3–2C_		*k*_flux–2C_
*MB* (%)	48	**–**	**–**			− 51		4
*σ*_*B*_ (%)	24	**–**	**–**			49		33
*σ*_*P*_ (%)	13	36	42			11		10
*σ*_*T*_ (%)	27	**–**	**–**			50		35
2C4K	*v*_B–2C_	*K*_1–2C_	*k*_2–2C_			*k*_3–2C_	*k*_4–2C_	*k*_flux–2C_
*MB* (%)	25	**–**	**–**			510	3173	914
*σ*_*B*_ (%)	20	**–**	**–**			748	3768	1122
*σ*_*P*_ (%)	11	16	36			65	44	40
*σ*_*T*_ (%)	23	**–**	**–**			751	3768	1123
3C5K	*v*_B_	*K*_1_	*k*_2_	*k*_3_	*k*_4_	*k*_5_		*k*_flux_
*MB* (%)	0	− 1	− 2	− 5	− 5	− 18		− 11
*σ*_*B*_ (%)	3	1	2	7	5	21		18
*σ*_*P*_ (%)	10	5	10	21	11	10		8
*σ*_*T*_ (%)	10	5	10	22	12	23		19
3C6K	*v*_B_	*K*_1_	*k*_2_	*k*_3_	*k*_4_	*k*_5_	*k*_6_	*k*_flux_
*MB* (%)	− 1	0	1	4	3	8	86	6
*σ*_*B*_ (%)	3	1	2	5	4	9	131	8
*σ*_*P*_ (%)	10	5	9	21	13	27	164	22
*σ*_*T*_ (%)	10	5	9	21	13	29	210	24

For parameters *ν*_B_, *K*_1_, *k*_2_, *k*_3_, and *k*_4_, mean biases and total uncertainties were similar for 3C5K and 3C6K model fits, regardless of which three-tissue model was used to represent the ground-truth. For the *k*_*5*_ and *k*_flux_ parameters, however, mean biases and total uncertainties were notably lower for 3C5K than for 3C6K model fits when the ground-truth was represented by the 3C5K model. Total uncertainties on 3C5K fits remained less than those on 3C6K fits even when the 3C6K model was used to represent the ground-truth, although in this circumstance 3C5K fit parameters had slightly higher mean biases than 3C6K fits.

Overall, the 3C5K model provided the most precise estimates of FMISO uptake kinetics according to statistical simulations, and the model’s accuracy was only surpassed by 3C6K when this reversible model was also considered to represent the ground-truth, despite the known irreversibility of FMISO binding.

### Correlations between *K*_1_ and *BF* parameters obtained from FMISO dPET and pCT

**Fig. 6:**
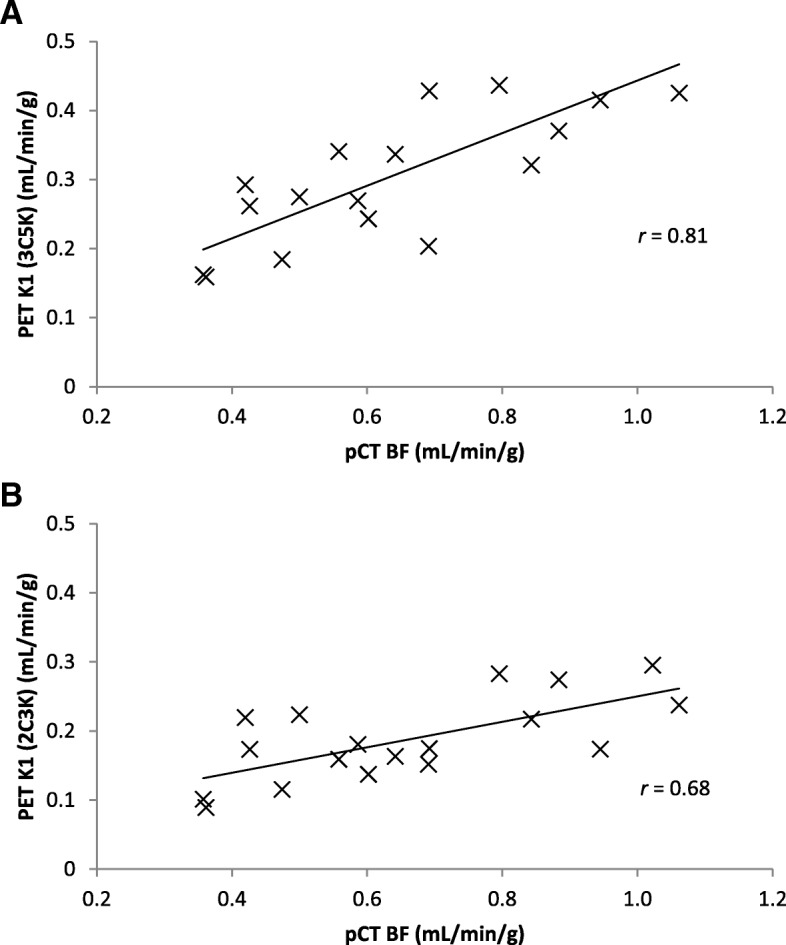
*K*_1_ values obtained from (**a**) 3C5K and (**b**) 2C3K compartment model fits to FMISO dPET data, plotted against *BF* values determined from pCT scans

**Fig. 7:**
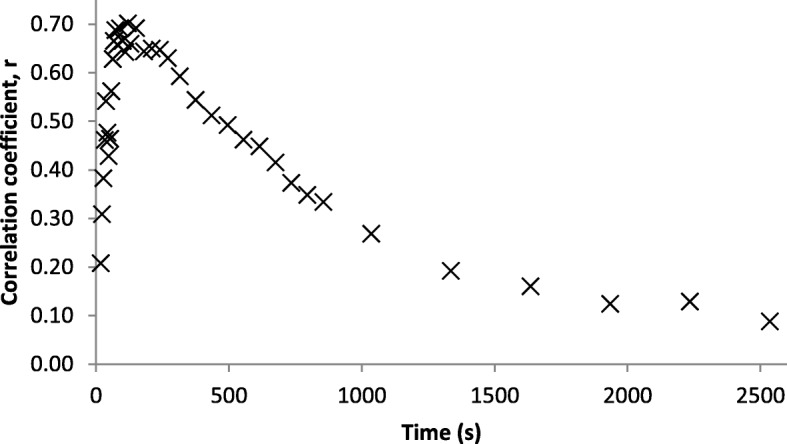
Pearson *r* coefficients of correlation between *BF* values and FMISO uptake at a range of times post-injection

Tumor *K*_1_ values obtained from fits of the 3C5K and 2C3K models to the FMISO dPET data are plotted in Fig. 6 against *BF* values independently obtained from pCT analysis. The 3C5K-based *K*_1_ values were strongly correlated with *BF* (Pearson *r* coefficient = 0.81), whereas 2C3K-based *K*_1_ values were less strongly correlated (*r* = 0.68). Pearson *r* coefficients of correlation between *BF* and static FMISO tumor uptake in frames collected at different times are plotted in Fig. 7, the maximum correlation (*r* = 0.70) being obtained at 2-min post-injection.

## Discussion

Whole tumor FMISO TACs obtained from dPET scans of advanced stage NSCLC patients were described better by an irreversible three-tissue compartment model, 3C5K, than by other compartment models we studied, according to information criterion and cross-validation scores, and statistical simulations. Total information criterion and cross-validation scores were much worse for simpler two-tissue compartment models and slightly worse for the reversible three-tissue model, 3C6K, and for four-tissue models whose additional complexity was unnecessary. In statistical simulation studies, total uncertainties calculated for fitted 3C5K model parameter values were consistently lower than those found for two-tissue compartment model fits, and a little lower than for 3C6K fits, even when the 3C6K model was used to represent the ground-truth.

For five of the measured whole tumor TACs, the 3C5K model fits did not pass a runs-test whereas the four-tissue compartment model fits did. For these particular TACs, we therefore carried out further statistical simulations, using 3C5K and 4C7K models as ground-truth. Even for these specific cases, fits of the 3C5K model provided more precise estimates of ground-truth kinetics values than did 4C7K fits, regardless of the ground-truth model used in the simulations.

A strong correlation (*r* = 0.81) was found between the *K*_1_ parameter values of the 3C5K model fits to the FMISO TACs and *BF* values independently obtained from pCT. The *K*_1_ values obtained from 2C3K model fits correlated less strongly (albeit not significantly less strongly) with *BF* (*r* = 0.68), lending weight to the results indicating that the 3C5K model describes whole tumor FMISO kinetics better than 2C3K. The *K*_*1*_ values obtained from 3C5K model fits were also more strongly correlated with *BF* than were whole tumor FMISO uptake values at times ranging from tracer injection to 4 h later (maximum correlation *r* = 0.70).

FMISO dPET imaging is used to measure hypoxia, and since hypoxia is related to perfusion, pCT perfusion scans are sometimes collected as well. Since *BF* is strongly correlated with the *K*_1_ values obtained from 3C5K model fits to FMISO dPET data, blood flow could potentially be estimated directly from the *K*_1_ values obtained from the FMISO images, rather than from pCT, thus saving time, money and the pCT radiation dose, and generating *BF* data over the 15 cm axial width of modern PET scanner fields-of-view, rather than 4-cm axial width typical of CT scanners used in cine-mode. Blood flow could also be estimated from FDG dPET data if available (not a hypoxia tracer), since *BF* values obtained from ^15^O-labeled water dPET studies have previously been shown to strongly correlate with parameter values obtained from model fits to the first 2 min of FDG dPET scans (*r* = 0.86) [33].

The gold-standard method for determining input functions is direct arterial line sampling. However, we have used image-derived input functions (IDIFs) calculated from mean tracer activity concentrations within volumes drawn in the descending aorta, both for patient comfort and safety, and because good agreement has been demonstrated between directly sampled input functions and IDIFs obtained from the descending aorta [11].

In this study, we have assessed the performance of PET kinetic models in terms of information criteria scores, cross-validation measures, statistical simulations, and strengths of correlations with an independent measure of perfusion. The imaging protocol used in this work is demanding, as we are currently investigating whether shorter protocols can provide adequate rate-constant estimates. In a recently opened study (Atovaquone as Tumor HypOxia Modifier, NCT02628080), surgically treated NSCLC patients are being imaged using dynamic FMISO PET prior to tumor excision, allowing us to compare FMISO images and parametric maps directly with maps of histopathology obtained from excised tumor slices.

## Conclusions

Time-courses of whole tumor FMISO uptake in patients with advanced stage NSCLC were described better by an irreversible three-tissue compartment model, 3C5K, than by other two-, three-, or four-tissue compartment models investigated. Fits of this model also provided the most precise estimates of FMISO uptake kinetics according to simulation studies. Further evidence for the utility of the 3C5K model was provided by the observation of a strong correlation (*r* = 0.81) between fitted values of its *K*_*1*_ parameter and blood flow values obtained independently from perfusion CT imaging, a stronger correlation than that between blood flow and *K*_1_ values obtained from 2C3K model fits, or between blood flow and tumor FMISO uptake in static scans taken at a range of times from immediately post-injection to 4 h later.

## Additional files

Additional file 1: **Table S1.** TAC-by-TAC Wald-Wolfowitz runs-test results for fits of the different models (ticks indicate runs-test passes) (DOCX 38 kb)

Additional file 2: **Table S2.** Individual AIC, BIC and MSEP scores for fits of the various models to each TAC. The lowest AIC, BIC, and MSEP scores have been underlined for each TAC, indicating the best model according to that measure (DOCX 46 kb)

## Abbreviations

2C3K: Irreversible two-tissue compartment model
3C5K: Irreversible three-tissue compartment model
AATH: Adiabatic Approximation to the Tissue Homogeneity
AIC: Akaike information criterion
BF: Blood flow
BIC: Bayesian information criterion
CT: Computed tomography
dPET: Dynamic PET
FDG: ^18^F-fluorodeoxyglucose
FMISO: ^18^F-fluoromisonidazole
IDIF: Image-derived input function
MB: Mean bias
MSEP: Mean of the squared error of prediction
NGF: Normalized gradient fields
NSCLC: Non-small cell lung cancer
pCT: Perfusion CT
PET: Positron emission tomography
RT: Radiotherapy
TACs: Time-activity-curves
VOI: Volume-of-interest
