# Supplementary material for: Whole tumor kinetics analysis of 18F-fluoromisonidazole dynamic PET scans of non-small cell lung cancer patients, and correlations with perfusion CT blood flow
Source: EJNMMI Res. 2018 Aug 1;8:73. doi: 10.1186/s13550-018-0430-4 (PMC6070455; doi:10.1186/s13550-018-0430-4)
Supplement: Supplementary file 1 — Table S1. TAC-by-TAC Wald-Wolfowitz runs-test results for fits of the different models (ticks indicate runs-test passes) (DOCX 38 kb) [file 13550_2018_430_MOESM1_ESM.docx]

| TAC | **2C3K** | **2C4K** | **3C5K** | **3C6K** | **4C7K** | **4C8K** |
| --- | --- | --- | --- | --- | --- | --- |
| 1 |  | ✓ | ✓ | ✓ | ✓ | ✓ |
| 2 |  |  | ✓ | ✓ | ✓ | ✓ |
| 3 |  |  |  |  | ✓ | ✓ |
| 4 |  |  |  |  | ✓ | ✓ |
| 5 |  |  | ✓ | ✓ | ✓ | ✓ |
| 6 |  |  | ✓ | ✓ | ✓ | ✓ |
| 7 |  |  | ✓ | ✓ | ✓ | ✓ |
| 8 |  |  | ✓ | ✓ | ✓ | ✓ |
| 9 |  | ✓ | ✓ | ✓ | ✓ | ✓ |
| 10 |  |  |  | ✓ | ✓ | ✓ |
| 11 |  |  | ✓ | ✓ | ✓ | ✓ |
| 12 |  |  | ✓ | ✓ | ✓ | ✓ |
| 13 |  |  | ✓ | ✓ | ✓ | ✓ |
| 14 |  |  | ✓ | ✓ | ✓ | ✓ |
| 15 |  |  | ✓ | ✓ | ✓ | ✓ |
| 16 |  |  | ✓ | ✓ | ✓ | ✓ |
| 17 |  |  |  |  | ✓ | ✓ |
| 18 |  |  |  |  | ✓ | ✓ |
| 19 |  | ✓ | ✓ | ✓ | ✓ | ✓ |
| 20 |  |  | ✓ | ✓ | ✓ | ✓ |
| 21 |  |  | ✓ | ✓ | ✓ | ✓ |
| 22 |  |  | ✓ | ✓ | ✓ | ✓ |
| 23 |  |  | ✓ | ✓ | ✓ | ✓ |
| 24 |  |  | ✓ | ✓ | ✓ | ✓ |
| 25 |  |  | ✓ | ✓ | ✓ | ✓ |
| 26 |  |  | ✓ | ✓ | ✓ | ✓ |
| 27 |  | ✓ | ✓ | ✓ | ✓ | ✓ |
| 28 |  | ✓ | ✓ | ✓ | ✓ | ✓ |
| 29 |  | ✓ | ✓ | ✓ | ✓ | ✓ |
| 30 |  |  | ✓ | ✓ | ✓ | ✓ |
|  |  |  |  |  |  |  |

Table S1 TAC-by-TAC Wald-Wolfowitz runs-test results for fits of the different models (ticks indicate runs-test passes).
