# Supplementary material for: Whole tumor kinetics analysis of 18F-fluoromisonidazole dynamic PET scans of non-small cell lung cancer patients, and correlations with perfusion CT blood flow
Source: EJNMMI Res. 2018 Aug 1;8:73. doi: 10.1186/s13550-018-0430-4 (PMC6070455; doi:10.1186/s13550-018-0430-4)
Supplement: Supplementary file 2 — Table S2. Individual AIC, BIC and MSEP scores for fits of the various models to each TAC. The lowest AIC, BIC, and MSEP scores have been underlined for each TAC, indicating the best model according to that measure (DOCX 46 kb) [file 13550_2018_430_MOESM2_ESM.docx]

| **Models** | **2C3K** | | | **2C4K** | | | **3C5K** | | | **3C6K** | | | **4C7K** | | | **4C8K** | | |
| --- | --- | --- | --- | --- | --- | --- | --- | --- | --- | --- | --- | --- | --- | --- | --- | --- | --- | --- |
| TAC | *AIC* | *BIC* | *MSEP* | *AIC* | *BIC* | *MSEP* | *AIC* | *BIC* | *MSEP* | *AIC* | *BIC* | *MSEP* | *AIC* | *BIC* | *MSEP* | *AIC* | *BIC* | *MSEP* |
| 1 | 170 | 170 | 7.4 | 49 | 57 | 2.4 | 34 | 43 | 0.52 | 37 | 47 | 0.52 | 41 | 50 | 0.54 | 44 | 54 | 0.52 |
| 2 | 540 | 550 | 8.2 | 310 | 320 | 1.6 | 51 | 60 | 0.70 | 55 | 64 | 0.70 | 58 | 68 | 0.71 | 62 | 71 | 0.70 |
| 3 | 580 | 590 | 17 | 260 | 270 | 5.0 | 78 | 87 | 1.8 | 81 | 90 | 3.1 | 56 | 65 | 1.5 | 59 | 69 | 3.1 |
| 4 | 260 | 270 | 14 | 88 | 96 | 4.6 | 62 | 70 | 4.2 | 65 | 74 | 4.1 | 56 | 65 | 4.1 | 59 | 69 | 4.1 |
| 5 | 720 | 730 | 4.4 | 490 | 500 | 4.7 | 52 | 61 | 0.36 | 49 | 59 | 0.39 | 52 | 62 | 0.37 | 56 | 66 | 0.37 |
| 6 | 800 | 800 | 2.5 | 380 | 390 | 1.7 | 52 | 61 | 0.15 | 54 | 63 | 0.15 | 57 | 67 | 0.15 | 61 | 71 | 0.15 |
| 7 | 230 | 230 | 4.3 | 150 | 160 | 4.8 | 51 | 60 | 1.1 | 55 | 64 | 1.8 | 58 | 67 | 1.3 | 62 | 71 | 1.5 |
| 8 | 350 | 350 | 4.4 | 130 | 140 | 2.5 | 52 | 61 | 0.49 | 52 | 62 | 0.63 | 59 | 69 | 0.57 | 59 | 69 | 0.59 |
| 9 | 140 | 150 | 23 | 48 | 55 | 2.2 | 29 | 37 | 1.1 | 32 | 41 | 1.9 | 33 | 42 | 1.6 | 36 | 45 | 1.8 |
| 10 | 590 | 590 | 6.2 | 200 | 210 | 2.3 | 100 | 120 | 1.7 | 54 | 64 | 0.46 | 58 | 68 | 1.3 | 56 | 66 | 1.3 |
| 11 | 860 | 870 | 19 | 700 | 710 | 18 | 52 | 61 | 0.65 | 55 | 65 | 0.78 | 59 | 69 | 0.85 | 63 | 73 | 0.77 |
| 12 | 400 | 410 | 2.6 | 260 | 270 | 4.8 | 52 | 61 | 0.51 | 37 | 46 | 0.51 | 41 | 51 | 0.51 | 44 | 54 | 0.54 |
| 13 | 1100 | 1100 | 11 | 650 | 660 | 4.7 | 51 | 60 | 0.29 | 52 | 62 | 0.29 | 58 | 67 | 0.34 | 59 | 69 | 0.31 |
| 14 | 1500 | 1500 | 6.4 | 970 | 980 | 5.3 | 51 | 60 | 0.14 | 54 | 63 | 0.17 | 50 | 60 | 0.17 | 54 | 64 | 0.56 |
| 15 | 250 | 260 | 21 | 190 | 200 | 9.6 | 51 | 59 | 1.3 | 54 | 63 | 1.3 | 57 | 66 | 1.3 | 61 | 70 | 1.3 |
| 16 | 510 | 520 | 15 | 350 | 360 | 10 | 51 | 60 | 0.60 | 54 | 63 | 1.0 | 45 | 55 | 0.4 | 49 | 58 | 1.1 |
| 17 | 1900 | 1900 | 6.1 | 1000 | 1100 | 4.0 | 80 | 89 | 0.18 | 83 | 93 | 0.21 | 57 | 67 | 0.15 | 60 | 70 | 0.18 |
| 18 | 490 | 490 | 5.8 | 410 | 410 | 5.7 | 75 | 84 | 0.73 | 72 | 82 | 0.70 | 57 | 67 | 0.75 | 58 | 68 | 0.72 |
| 19 | 82 | 89 | 4.6 | 50 | 58 | 3.9 | 24 | 33 | 0.48 | 28 | 37 | 0.48 | 31 | 41 | 0.48 | 34 | 45 | 0.48 |
| 20 | 120 | 120 | 3.7 | 100 | 110 | 2.7 | 52 | 61 | 0.82 | 53 | 62 | 0.85 | 71 | 81 | 0.84 | 73 | 84 | 0.90 |
| 21 | 330 | 330 | 1.7 | 300 | 310 | 3.1 | 51 | 60 | 0.74 | 54 | 64 | 0.74 | 58 | 67 | 0.74 | 61 | 71 | 0.74 |
| 22 | 370 | 380 | 3.8 | 260 | 270 | 3.8 | 52 | 61 | 0.33 | 52 | 62 | 0.34 | 55 | 65 | 0.34 | 58 | 69 | 0.34 |
| 23 | 130 | 140 | 1.6 | 130 | 140 | 1.8 | 52 | 61 | 0.61 | 61 | 71 | 0.61 | 59 | 69 | 0.61 | 62 | 72 | 0.61 |
| 24 | 510 | 520 | 2.9 | 460 | 470 | 4.9 | 52 | 61 | 0.31 | 55 | 65 | 0.31 | 59 | 69 | 0.31 | 62 | 72 | 0.31 |
| 25 | 220 | 230 | 2.5 | 200 | 210 | 2.4 | 52 | 61 | 0.59 | 55 | 65 | 0.59 | 59 | 69 | 0.59 | 62 | 72 | 0.59 |
| 26 | 420 | 430 | 3.8 | 210 | 220 | 4.3 | 51 | 60 | 0.57 | 54 | 65 | 0.57 | 58 | 69 | 0.57 | 61 | 72 | 0.57 |
| 27 | 67 | 74 | 2.6 | 50 | 58 | 2.7 | 31 | 40 | 0.89 | 38 | 47 | 0.89 | 37 | 47 | 0.90 | 41 | 51 | 0.92 |
| 28 | 160 | 170 | 5.1 | 50 | 58 | 3.2 | 42 | 51 | 1.4 | 46 | 55 | 1.4 | 49 | 59 | 1.4 | 52 | 63 | 1.4 |
| 29 | 106 | 113 | 3.9 | 50 | 58 | 1.5 | 31 | 40 | 0.60 | 34 | 44 | 0.69 | 37 | 47 | 0.66 | 41 | 51 | 0.63 |
| 30 | 84 | 91 | 4.5 | 50 | 58 | 2.3 | 26 | 34 | 0.65 | 29 | 38 | 0.68 | 32 | 42 | 0.66 | 35 | 46 | 0.66 |
|  |  | | |  | | |  | | |  | | |  | | |  | | |

Table S2 Individual AIC, BIC and MSEP scores for fits of the various models to each TAC. The lowest AIC, BIC and MSEP scores have been underlined for each TAC, indicating the best model according to that measure.
